# Case Report: Rapid renal response to venetoclax monotherapy in a CLL patient with secondary membranous glomerulonephritis

**DOI:** 10.3389/fonc.2023.1108994

**Published:** 2023-04-21

**Authors:** Ester Lovato, Concetta Gangemi, Mauro Krampera, Carlo Visco, Isacco Ferrarini

**Affiliations:** ^1^ Section of Hematology, Department of Medicine, University of Verona, Verona, Italy; ^2^ Division of Nephrology and Dialysis, University Hospital of Verona, Verona, Italy

**Keywords:** chronic lymphocytic leukemia, venetoclax, membranous glomerulonephritis, autoimmune complications, nephrotic syndrome

## Abstract

Membranous glomerulonephritis (MGN) is a rare extra-hematological autoimmune complication of chronic lymphocytic leukemia (CLL), clinically characterized by nephrotic-range proteinuria and, less frequently, renal failure. Because of the rarity of this condition, there is no standardized treatment. Chlorambucil and fludarabine-based regimens, possibly combined with rituximab, have been historically the most frequent therapeutic approaches, with renal response obtained in about two-third of the patients. However, responses are often transient and partial. Here we describe the first patient with rituximab-refractory, CLL-related MGN successfully treated with the Bcl-2 antagonist venetoclax. Nephrotic syndrome resolved as soon as three months after venetoclax initiation, with no unexpected toxicities. At the last follow-up, 17 months after venetoclax start, renal response persists, with proteinuria below 0.5 g/24 hours. This case suggests that targeted agents, particularly Bcl-2 antagonists, might be suitable options for patients with renal autoimmune disorders arising in the context of CLL.

## Introduction

Chronic lymphocytic leukemia (CLL), the most common type of leukemia in western countries, derives from the clonal expansion of mature B lymphocytes within bone marrow, lymph nodes and other hematopoietic organs. Although it usually follows an indolent clinical course, a significant fraction of patients eventually requires treatment, most often due to progressive bone marrow failure or bulky adenopathy (1). In addition, 10 to 25% of CLL cases display autoimmune complications during the disease course, most commonly autoimmune hemolytic anemia and immune thrombocytopenia (2). Non hematological autoimmune manifestations, such as pemphigus, thyroiditis, vasculitis, rheumatoid arthritis, and glomerulonephritis, are less frequent, yet sometimes highly impacting on patients’ morbidity and treatment indications.

Glomerulonephritis encompasses a range of autoimmune disorders causing inflammation within the glomerulus and other renal compartments (3). Glomerulonephritis can be either idiopathic or secondary to autoimmune, infectious, malignant or metabolic diseases. Clinical presentation varies from asymptomatic urinary abnormalities, to nephrotic syndrome, or rapidly progressing renal failure (3). In most cases, glomerulonephritis is considered a progressive condition leading to glomerular sclerosis and tubulointerstitial fibrosis, with a decline of glomerular filtration rate over time. Indeed, it is the cause of 15% of end-stage renal diseases in the United States. Timely therapeutic intervention is required to preserve glomerular function and reduce the risk of glomerular fibrosis and end-stage disease progression (3).

In a retrospective analysis published in 2015, 49 of 4024 patients (1.2%) affected by CLL or monoclonal B-cell lymphocytosis (MBL) underwent kidney biopsy for renal failure or nephrotic syndrome (4). Thirty-five out of 49 biopsies (71%) displayed variable degree of CLL infiltration, and 21 (43%) were compatible with immune-mediated glomerulonephritis. The most common glomerulopathy described in association with CLL is membranoproliferative glomerulonephritis (MPGN). Membranous glomerulonephritis (MGN) is the second most common renal autoimmune manifestation arising in the context of CLL (4). It is characterized by thickening of the glomerular basement membrane, usually involving the whole glomerulus and diffuse to the majority of glomeruli (5). Anti-phospholipase A2 receptor (PLA2R) antibodies, typically encountered in idiopathic MGN, are usually negative in secondary forms (5). Treatment recommendations for CLL-related MGN are mainly based on a few case series and include steroids, alkylating agents, and anti-CD20 antibodies, with no standardized approach (4). Moreover, only few data are available about the potential role of ibrutinib and venetoclax, two widely used CLL-directed targeted agents (6), in the management of autoimmune renal complications.

Here we report for the first time on a CLL patient with secondary, rituximab-refractory MGN who showed rapid and durable renal response to venetoclax monotherapy.

## Case description

A 62-years old man was diagnosed with Rai I/Binet B CLL in June 2012. He had diffused lymphadenopathy causing right leg deep venous thrombosis and lymphedema. His blood cell count showed leukocytosis (27,800/μL, 82% lymphocytes; normal values [nv] 4,300-10,000/μL), with mild anemia (hemoglobin 11 g/dL; nv 13.5-17 g/dL) and normal platelet count (223,000/μL; nv 150,000-400,000/μL). Peripheral blood phenotyping was consistent with CLL, and characterized by the expression of CD5 and CD23, while FMC7 and CD38 were negative. CD49d was partially expressed. Bone marrow biopsy revealed a diffuse monomorphic infiltration of mature-appearing lymphocytes (80% of bone marrow cellularity), confirming the diagnosis of CLL. The patient had history of arterial hypertension, prostate adenocarcinoma (surgically removed, in regular follow-up), and pulmonary emphysema, with no sign of renal disease. Due to progressive adenopathy, he was treated with 4 cycles of Bendamustine-Rituximab in 2012-2013, obtaining partial remission with improvement of blood counts and clear-cut reduction of adenopathy.

**Figure 1:**
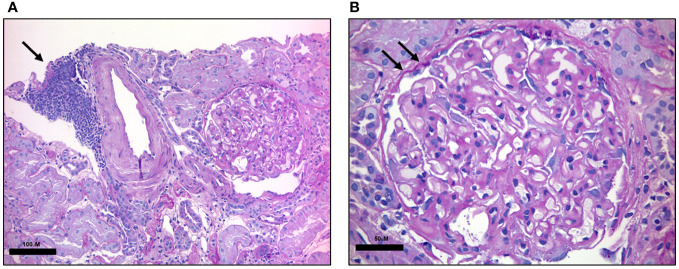
Renal histology. PAS-stained renal biopsy. **(A)** Low magnification shows a dense lymphocytic infiltrate composed of small monotonous lymphocytes in aggregate (black arrow). Scale bar: 100 μM **(B)** High-magnification highlights renal glomerulus with thickening of the basal membrane (black arrows). Scale bar: 50 μM.

In 2015 proteinuria was noted (urinary protein excretion 2.75 g/24h, nv <0.15 g/24h), without any clinical symptoms. Proteinuria progressively increased over the following years, reaching the nephrotic range on March 2017 (4 g/24 hours), with normal creatinine (0.55 mg/dL, nv 0.59-1.29 mg/dL), elevated cholesterol (total cholesterol 259 mg/dL nv <200 mg/dL, HDL 51 mg/dL nv >50 mg/dL, LDL 207 mg/dL nv <160 mg/dL), normal triglycerides (117 mg/dL, nv <150 mg/dL), and low serum albumine (22.7 g/L, nv 35-50 g/L). The patient was then referred to the Nephrology Division to perform a percutaneous kidney biopsy aiming at clarifying the underlying pathological process. Histology on 12 glomeruli showed thickening of the glomerular basement membrane with limited CLL infiltrate (**Figures 1A, B**). Immunofluorescent staining was negative for IgG, IgA, IgM, C1q, kappa or lambda chains deposition, and revealed only focal segmental deposition of the third complement component (C3). Serological tests for anti-nuclear antibodies, perinuclear Anti-Neutrophil Cytoplasmic Antibody, cytoplasmic Anti-Neutrophil Cytoplasmic Antibody, HIV, hepatitis B and C as well as anti-PLA2R were negative. No monoclonal peak was detected in either serum or urine electrophoresis. Bence Jones proteinuria was undetectable. The serum third and fourth component of complement (C3, C4) were within the normal range. A diagnosis of MGN was made, combined with parcel renal localization of CLL.

**Figure 2:**
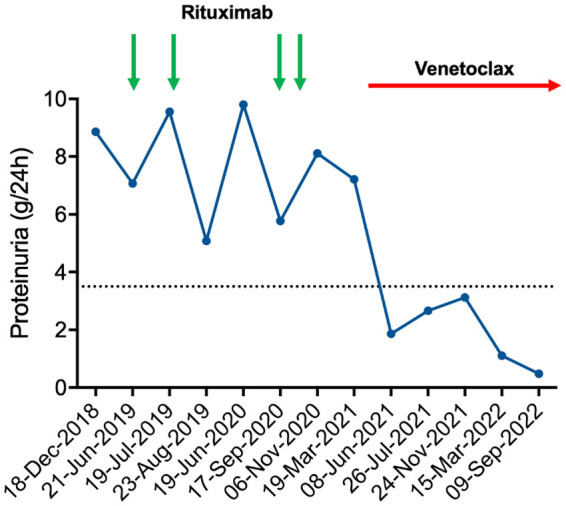
Proteinuria time course. Schema of patient’s proteinuria in relation to treatments administered. Rituximab was administered at 700 mg each dose. Venetoclax was started according to the standard ramp-up schedule. As evident by the graph, proteinuria had a partially remitting-relapsing course during rituximab treatment, with all values persistently above the nephrotic threshold (dashed line). By contrast, venetoclax led to a rapid and durable reduction of proteinuria that fell below the nephrotic threshold.

The patient was in good general conditions, with long-lasting response to the first line treatment and no stringent hematological criteria for further CLL-directed therapy. Peripheral blood counts showed hemoglobin 11.7 g/dL, platelets 172,000/mmc, and leukocytes 68,470/mmc with neutrophils 5,540/mmc. At the CT scan of September 2018, multiple small lymphadenopathies on both sides of diaphragm (max 23 mm) were detected, together with moderate asymptomatic splenomegaly (15.5 cm in largest diameter). Biological assessment showed mutated IGHV, wild-type *TP53*, normal karyotype, and no alterations on fluorescent *in situ* hybridization (FISH) analysis (del11q, del13q, del17p, and trisomy 12 were tested). Due to further worsening of nephrotic syndrome, with proteinuria reaching 7 g/24h in June 2019, Rituximab treatment was started (**Figure 2**). Two monthly doses of Rituximab at 700 mg were administered without significant toxicity. However, there was no sign of biochemical improvement with persistently high values of proteinuria. In September 2020, two further doses of Rituximab were given, with no sign of renal response (proteinuria 8.11 g/24h in November 2020). Rituximab treatment led to moderate CLL tumor burden reduction with leucocyte count decreasing from 68,470/μL to 47,460/μL at the end of the treatment, and spleen largest diameter reduction from 15.5 to 14 cm.

Owing to rituximab refractoriness, we set out to treat the underlying CLL in an attempt to improve the renal damage. Based on pre-clinical evidence showing that Bcl-2 inhibition might be beneficial in immune-mediated renal disorders (7), second line therapy with venetoclax was started on April 2021, following the standard ramp-up schedule (20 mg once daily for the first week, 50 mg once daily for the second week, 100 mg once daily for the third week, 200 mg once daily for the fourth week and then 400 mg once daily continuously) (8). The patient was classified as having a medium risk of tumor lysis syndrome (TLS) based on his lymphocyte counts and lymph node diameters. He received allopurinol as anti-uricemic prophylaxis and intravenous hydration. Biochemical blood tests were closely monitored during the ramp-up phase. Since there was no sign of TLS, hospitalization was not needed. Decline of urinary protein excretion was observed as soon as 12 weeks after venetoclax initiation, with proteinuria falling from 7.21 g/24h (March 2021) to 1.86 g/24h (June 2021) (**Figure 2**). No toxicities were recorded, apart from grade 3 neutropenia that led to 25% venetoclax dose reduction after 2 months of treatment. Lymphocyte count normalized after the first 2 weeks of venetoclax treatment (from 26,410/mmc to 2,440/mmc). Hemoglobin improved after the first 4 months of treatment (from 10.2 to 12.7 g/dL) as well. At the last follow up, 17 months after venetoclax start, the patient is still on therapy and his proteinuria remains below the nephrotic range (0.48 g/24h on September 2022), confirming a durable partial remission of MGN-related nephrotic syndrome.

## Discussion and literature review

MGN is a rare but well-recognized CLL-related autoimmune renal complication, most commonly arising in the context of active CLL. To the best of our knowledge, 28 cases of histologically proven, CLL-related MGN have been reported in the literature so far (4, 9–25) (**Table 1**). Twenty out of 22 cases (90.9%) with available data were clinically characterized by nephrotic-range proteinuria, while 52.9% had elevated serum creatinine (≥1.3 mg/dL). Only 2 cases were associated to the presence of a monoclonal spike (IgM class), arguing against a pathogenetic role for monoclonal proteins. All evaluable patients tested negative for anti-PLA2R antibodies, further indicating they are CLL-related, rather than idiopathic, MGN cases. Immunofluorescence on renal biopsies detected granular subepithelial deposits of IgG and C3 fraction in 95.8% of cases, whereas CLL infiltration was documented in 83.3% of evaluable cases. Because up to 90% of CLL patients with no previous kidney dysfunction have some degree of renal lymphoid infiltration on autopsy (26), it is unlikely that renal damage is sustained directly by the CLL infiltrate. Although the exact pathophysiology of secondary MGN remains undefined, the formation of antibodies against CLL antigens that are immunologically similar to podocyte antigens might be responsible for the immune-mediated attack to the glomerular membrane (27). Treatment strategies for CLL-related MGN were widely heterogeneous and changed over time. Chlorambucil-based regimens (7/18 evaluable patients) were more frequently administered until 1990s, while fludarabine-based treatments were preferred from 1999 onward. Rituximab was given to 4 patients, in combination with either fludarabine-cyclophosphamide, bendamustine, or chlorambucil. None of these patients received small molecules as treatment of their MGN. Fourteen out of 18 treated patients had some degree of renal response, often with a concomitant amelioration of their underlying CLL. However, in 5 of them renal improvement was either transient or partial, suggesting that more effective and less toxic strategies are needed for this rare condition. BTK inhibitors and Bcl-2 antagonists are two classes of targeted agents that have revolutionized the treatment paradigms of CLL in recent years (6). However, their efficacy in treating hematological and extra-hematological autoimmune complications is still under clinical investigation. In a recent study including 104 CLL patients affected by autoimmune cytopenia and treated with a targeted agent, 80% of them experienced an improvement or even resolution of the autoimmune phenomena during treatment (28), indicating that new drugs can be suitable options to control pre-existing, CLL-related autoimmune conditions. Wang and colleagues recently described two cases of CLL-related MPGN treated with the BTK inhibitor ibrutinib who achieved complete renal recovery (24), further suggesting that targeted agents might have immune-regulatory effects potentially beneficial in extra-hematological immune complications.

**Table 1:** CLL-related MGN cases reported in the literature to date.

Case	Reference	At diagnosis of MGN								Glomerular lesions				Treatment	Outcome	
		age/sex	creatinine (mg/dl)	proteinuria (g/24h)	monoclonal protein	complement	PLA2R	prior duration of CLL	prior CLL therapy	light microscopy	IF	electron microscopy	CLL infiltrate		nephrotic syndrome	CLL
1	(9)	64/M	NA	NA	NA	NA	NA	S	None	MGN	NA	NA	NA	Chl	Partial response	NA
2	(10)	61/M	NA	NA	None	NA	NA	NA	NA	MGN	IgG, C3, C1q	NA	NA	Chl	Improve	Improve
3	(11)	69/M	Normal	8.5	None	→	NA	5y	None	MGN	IgG, C3	NA	Yes	NA	NA	NA
4	(12)	57/F	NA	NA	None	C3, C4→ CH50↓	NA	NA	NA	Atypical MGN	IgG, k, complement	Subepithelial deposits	No	Chl + PDN	Improve	Improve
5		75/M	NA	NA	IgM κ	C3→ CH50, C1q, C4↓	NA	NA	NA	Atypical MGN	IgG, IgM, κ, λ, complement	Subepithelial deposits	Yes	Chl	Improve	Improve
6	(13)	55/M	NA	≥3	None	CH50↓	NA	S	None	Atypical MGN	IgG, C3, κ	Fibrillary deposits	Yes	CHOP + Chl	Partial response	NA
7	(14)	82/F	1.3	5-8	None	C3, C4, C1q →	NA	17y	Chl + PDN	MGN	NA	Fibrillary deposits	No	CTX + PDN, CsA	NR Improve	NR, Improve
8	(15)	NA/F	Normal	6.7	None	NA	NA	4y	Chl	MGN	NA	Subepithelial deposits	NA	CTX + PDN, Fludarabine	NR Improve	NR Improve
9	(16)	58/F	3.8	19.2	None	NA	NA	2y	None	MGN	NA	NA	NA	Fludarabine	Improve	Improve
10	(17)	66/F	1.18	5-8	None	C3, C4, CH50, C1q →	NA	S	None	MGN	IgG, C3	Subepithelial fibrillary deposits	Yes	CTX + PDN	Transient improve	Improve
11	(18)	72/M	0.8	5	None	NA	NA	6m	Chl + PDN	MGN	IgG, C3	Subepithelial deposits	Yes	Chl + PDN	Partial response	Stable
12	(19)	73/M	3.2	4.3	None	C3, C4 →	NA	NA	None	MGN	IgG, C3, C1q, κ, λ	NA	Yes	COP	Improve	Improve
13	(20)	56/M	2.4	13	NA	NA	NA	NA	NA	MGN (focal segmental sclerosis)	IgG, C3, κ, λ	NA	Yes	NA	NA	NA
14	(21)	74/M	1.7	7	None	C3, C4 →	NA	6y	None	MGN	IgG, IgM, C3, C1q and λ	NA	Yes	Fludarabine	Improve	Improve
15	(4)	NA	Elevated	<3	NA	NA	NA	NA	NA	MGN	NA	NA	Yes	NA	NA	NA
16		NA	Normal	≥3	NA	NA	NA	NA	NA	MGN	NA	NA	Yes	NA	NA	NA
17	(22)	67/F	1	9.6	None	C4 →	Neg (biopsy)	NA	NA	MGN (focal proliferation)	IgG, C3, κ	Subepithelial deposits	NA	NA	NA	NA
18		67/F	2.3	NA	None	C4 →	Neg (biopsy)	NA	NA	MGN (focal crescents)	IgG, C3, κ	Subepithelial deposits	NA	NA	NA	NA
19		64/F	1.4	7.4	None	C4 →	Neg (biopsy)	NA	NA	MGN	IgG, C3, λ	Subepithelial deposits	NA	NA	NA	NA
20		64/M	1	5	None	C4 →	Neg (biopsy)	NA	NA	MGN	IgG, C3, C1q, κ	Subepithelial deposits	NA	NA	NA	NA
21		63/M	NA	8	None	C4 →	Neg (biopsy)	NA	NA	MGN (focal proliferation)	IgG, C3, C1q, λ	Subepithelial deposits	NA	NA	NA	NA
22	(23)	61/M	NA	≥3	NA	NA	Neg	3m	None	MGN	IgG, C3	NA	Yes	FCR	Improve	Improve
23		79/M	NA	≥3	NA	NA	Neg	NA	None	MGN	IgG, IgM, C3	NA	No	None	Partial remission	Stable
24		73/F	NA	≥3	None	NA	Neg	8y	None	MGN	IgG, C3	NA	Yes	BR	Improve	Improve
25		72/M	NA	≥3	None	NA	Neg	S	None	MGN	IgG, C3	NA	Yes	Rituximab + Chl	NR	Improve
26	(24)	67/M	1.34	10.35	IgMλ	C3↓ C4→	NA	S	NA	MGN	IgG, C3, C1q	Subepithelial deposits	Yes	CTX, CsA	NR	Stable
27	(25)	59/M	1.14	NA	None	C3, C4 →	Neg (serum Ab)	1y	None	MGN	IgG, κ	NA	NA	FCR	Improve	No need to treat
28	Present case	67/M	0.49	4	None	C3, C4 →	Neg (serum Ab)	5y	BR	MGN	C3	Subepithelial deposits	Yes	Venetoclax	Improve	No need to treat

IF, immunofluorescence; NA, not available; NR, no response; S, simultaneous diagnosis; Chl, chlorambucil; →, normal; ↓, low; PDN, prednisone; CHOP, cyclophosphamide, adriamycin, vincristine and prednisone; CTX, cyclophosphamide; CsA, cyclosporine A; COP, cyclophosphamide, vincristine and prednisone; BR, bendamustine and rituximab; FCR, fludarabine, cyclophosphamide, rituximab.

Our case recapitulates the main features of CLL-related MGN. The patient had nephrotic-range proteinuria associated with elevated cholesterol, low serum albumin, and normal creatinine. Kidney biopsy demonstrated thickening of the glomerular basement membrane with C3 deposition, together with limited CLL infiltration. As Rituximab did not significantly improve the renal damage and a delayed response to this treatment was considered unlikely, the patient was started on the Bcl-2 antagonist venetoclax, which led to a rapid and sustained reduction of his proteinuria without any unexpected toxicity. In mouse models of autoimmune nephritis, treatment with venetoclax proved effective at prolonging animal survival and preventing the onset of tubule-interstitial inflammation and proteinuria (7). These pre-clinical data, together with our in-human experience, highlight Bcl-2 as a possible therapeutic target to be explored in MGN as well as in other idiopathic and secondary autoimmune renal disorders. Given the concerns related to the administration of venetoclax ramp-up in patients with impaired creatinine clearance, we consider Bcl-2 antagonism as an attractive option when nephrotic syndrome, rather than renal failure, dominates the clinical picture.

## Conclusions

To the best of our knowledge, we describe here the first case of CLL-related MGN successfully treated with venetoclax as single agent. In this patient, venetoclax largely outperformed rituximab at reducing proteinuria, and rapidly led to clinical remission of nephrotic syndrome. The depth and durability of renal response induced by venetoclax suggest that Bcl-2 antagonism might be a valuable therapeutic option for the management of CLL-related, and perhaps even idiopathic, autoimmune renal disorders.

## Data availability statement

The original contributions presented in the study are included in the article/supplementary material. Further inquiries can be directed to the corresponding author.

## Ethics statement

Written informed consent was obtained from the individual(s) for the publication of any potentially identifiable images or data included in this article.

## Author contributions

EL and IF performed the literature review and wrote the manuscript. CG provided scientific insights and renal biopsy images. MK and CV provided scientific insights and revised the manuscript. All authors contributed to the article and approved the submitted version.
